# Haplotype-Contained PCR Products Analysis by Sequencing with Selective Restriction of Primer Extension

**DOI:** 10.1155/2017/1397902

**Published:** 2017-12-10

**Authors:** Liu Wang, Pengfeng Xiao

**Affiliations:** State Key Laboratory of Bioelectronics, National Demonstration Center for Experimental Biomedical Engineering Education, School of Biological Science and Medical Engineering, Southeast University, Nanjing 210096, China

## Abstract

We develop a strategy for haplotype analysis of PCR products that contained two adjacent heterozygous loci using sequencing with specific primers, allele-specific primers, and ddNTP-blocked primers. To validate its feasibility, two sets of PCR products, including two adjacent heterozygous SNPs,* UGT1A1*⁎6 (rs4148323) and* UGT1A1*⁎28 (rs8175347), and two adjacent heterozygous SNPs, K1637K (rs11176013) and S1647T (rs11564148), were analyzed. Haplotypes of PCR products, including* UGT1A1*⁎6 and* UGT1A1*⁎28, were successfully analyzed by Sanger sequencing with allele-specific primers. Also, haplotypes of PCR products, including K1637K and S1647T, could not be determined by Sanger sequencing with allele-specific primers but were successfully analyzed by pyrosequencing with ddNTP-blocked primers. As a result, this method is able to effectively haplotype two adjacent heterozygous PCR products. It is simple, fast, and irrespective of short read length of pyrosequencing. Overall, we fully hope it will provide a new promising technology to identify haplotypes of conventional PCR products in clinical samples.

## 1. Introduction

Although research based on individual single nucleotide polymorphisms (SNPs) may lead to significant findings, methods based on haplotypes comprising multiple SNPs on the same inherited chromosome may provide additional power for mapping disease genes. Such insights may provide information essential for understanding human evolution and also for identifying cis-interactions between two or more causal variants [1–3]. However, the existing experimental methods for haplotyping, such as single-molecule dilution [4], cloning [5], and allele-specific polymerase chain reaction (AS-PCR) [6], are more laborious, complex, and expensive than genotyping [7]. In fact, if either of the two adjacent loci is homozygous, the haplotypes can be determined just by genotyping. For example, assume that the first locus has alleles A or T and the second locus G or C. Both loci, then, have three possible genotypes: (AA, AT, and TT) and (GG, GC, and CC), respectively. For a given individual, there are nine possible haplotypes at these two loci, as shown in Table 1. The haplotypes are determined when individuals are homozygous at one or both loci. When individuals are heterozygous at both loci, accounting for 1/9 of the total amount, the haplotypes are ambiguous—meaning that there is a need for further analysis. After genotyping has been performed, it is imperative to establish a simple and inexpensive method for the direct haplotyping of PCR products containing two adjacent heterozygous loci.

Pyrosequencing is an effective SNP typing method that can be used not only for SNP genotyping but also for quantitative analysis of heterogeneous DNA samples [8]. Thus, quantitative haplotypes are determined after SNPs have been genotyped when individuals are homozygous at one or both loci. The PCR products of individuals that are heterozygous at both loci can be used for haplotyping. Recently, we proposed a quantitative haplotyping of PCR products based on a nonsynchronous pyrosequencing strategy [9]. This approach can accurately infer haplotypes of unrestricted conventional PCR products, including both adjacent heterozygous loci, by a single sequencing run. However, this method has two disadvantages. One is that the procedure remains tedious; four different haplotypes must be obtained at the beginning and used for determining coefficients that also may change if the operational conditions are unstable. The other is that the read length of the sequencing is limited and only suitable for the analysis of short segment sequences.

As is well known, the PCR products comprising a homozygous locus can be obtained by AS-PCR and then used for haplotyping by sequencing, or mass spectrometry and other methods [6]. Based on traditional AS-PCR, this paper has developed a simple, fast, and cost-effective strategy for analyzing the haplotypes of unrestricted PCR products. First, PCR products heterozygous at both loci are sequenced with allele-specific primers. Then, for the samples that cannot be haplotyped with the allele-specific primer, the sequencing primers are selectively blocked with dideoxyribonucleoside triphosphate (ddNTP) to restrict the designated primer extension, and the haplotypes of the PCR products are identified by sequencing.

In this study, K1637K and S1647T related to Parkinson's disease [10] and* UGT1A1∗*6 and* UGT1A1∗*28 related to Gilbert's syndrome [11] were selected as experimental sites. The former two sites are located 27 bp apart in exon 34 of the leucine-rich repeat kinase 2* (LRRK2)* gene, and the latter are 268 bp apart in exon 1 of the UDP-glucuronosyltransferase 1A1* (UGT1A1)* gene. Here we present our novel approach that will provide a simple, direct, and inexpensive approach for detecting haplotypes of unrestricted conventional PCR products from natural samples in clinical diagnosis.

## 2. Materials and Methods

### 2.1. Reagents

ddNTPs were purchased from Roche Diagnostics (Mannheim, Germany), and exo^−^ Klenow (5000 U/ml) was purchased from Thermo Scientific (Shanghai, China). Inorganic pyrophosphatase (40 U/ml) and Sequenase version 2.0 T7 DNA polymerase (13 U/ml) were obtained from USB Corporation (Cleveland, OH, USA). Pfu PCR MasterMix (2x) was purchased from Solarbio (Beijing, China). Streptavidin-coated Sepharose™ High-Performance beads were purchased from GE Healthcare (Uppsala, Sweden). PyroMark Q24 Advanced Reagents were purchased from Qiagen (Hilden, Germany).

### 2.2. Primers and Target Sequences

All the oligonucleotides were purchased from RuiZhen (Nanjing, China). The oligonucleotides (Table 2) related to K1637K and S1647T were synthesized to verify the feasibility of the ddNTP blocking efficiency.

The sequences of the PCR primers and SNP-typing primers are listed in Table 3. DNA fragments from the* LRRK2* and* UGT1A1* genes were amplified using the PCR primers listed in Table 3 and used as templates for genotyping and haplotyping. K1637K and S1647T are on the same DNA fragment amplified by the primer pair TF and TR.* UGT1A1∗*6 and* UGT1A1∗*28 are on the same DNA fragment amplified by the primer pair GF and GR. To capture a single strand of DNA with Streptavidin beads, the 5′ ends of primers TR and GF were modified by biotin. All the oligonucleotides were purified by high-performance liquid chromatography.

### 2.3. DNA Extraction

Twenty blood samples were obtained from the Southeast University Zhongda Hospital (Nanjing, China). Genomic DNA was extracted from 200 *μ*L of whole blood using a QIAamp DNA Mini Kit (Qiagen, Valencia, CA, USA) following the manufacturer's instructions. All research procedures were approved by the Ethics Committee of the Southeast University Zhongda Hospital (Nanjing, China).

### 2.4. PCR Amplification

The template DNA was obtained by PCR with primer pair TF and TR from human genome DNA to obtain 148-bp fragments of the* LRRK2* gene. The amplification was performed with the following protocol: initial denaturing at 94°C for 3 min, followed by 35 thermal reaction cycles (94°C for 30 s, 60.2°C for 30 s, and 72°C for 30 s); after the thermal cycle reaction, a final extension at 72°C for 5 min was performed to ensure the complete extension of the amplified DNA fragment. The template DNA was obtained with primers GF and GR to obtain 432-bp fragments of the* UGT1A1* gene. The cycling conditions were as follows: 94°C for 3 min, 32 cycles of 94°C for 30 s, 54.2°C for 45 s, and 72°C for 45 s and then 72°C for 10 min.

A total of 1.5 ng of genomic DNA was used in a 50 *μ*L reaction, containing 0.4 *μ*M each of the forward and reverse primers, 2x Pfu PCR MasterMix (Solarbio, Beijing, China). PCR products were verified by electrophoresis with 2% agarose gels.

### 2.5. Sanger Sequencing with Allele-Specific Primers

Sanger sequencing was performed by Invitrogen (Shanghai, China) using the allele-specific primers SP6 and SP7 for haplotyping SNP loci in* UGT1A1*.

### 2.6. Pyrosequencing with ddNTP-Blocked Primers

*Template DNA Preparation*. In addition to synthesized single-stranded templates T1 and T2, selected natural DNA samples were used as PCR templates and amplified to obtain specific PCR products. Biotinylated DNA strands were immobilized on Sepharose beads. The immobilization was performed as follows: for each sample, the PCR product (10 *μ*L) was transferred into a new PCR tube with 40 *μ*L binding buffer, 1 *μ*L beads, and 29 *μ*L water and mixed at room temperature, 3.29 g for 15 min (MixMate, Eppendorf). Single-stranded DNA was obtained by a standard protocol using a Vacuum Prep Tool (Biotage AB, Uppsala, Sweden).

*Selectively Blocking the Sequencing Primers*. Single-stranded template DNA was hybridized to 10 pmol of the corresponding sequencing primer in 20 *μ*L PyroMark Annealing Buffer at 80°C for 5 min (ThermoStat™ C, Eppendorf).

Exo^−^ Klenow was applied for blocking the primers with ddNTP. 1 *μ*L of 500 *μ*M corresponding ddNTP and 2.5 units of exo^−^ Klenow (5000 U/mL) were added to the mixture and incubated at 37°C for 40 min and then placed into PyroMark Q24 Advanced instrument (Qiagen, Hilden, Germany).

Sequenase version 2.0 T7 DNA polymerase was also applied for blocking the primers with ddNTP. 1 *μ*L of 500 *μ*M corresponding ddNTP, 3.25 units Sequenase version 2.0 T7 DNA polymerase (13 U/mL), and 0.01 units inorganic pyrophosphatase (40 U/mL) were added to the mixture and incubated at 37°C for 10 min and then placed into PyroMark Q24 Advanced instrument (Qiagen, Hilden, Germany).

*Pyrosequencing*. Pyrosequencing was carried out on PyroMark Q24 Advanced instrument and PyroMark Q24 Advanced software (Qiagen, Hilden, Germany). The pyrograms were analyzed, and peak heights were obtained using the AQ mode of the PyroMark Q24 Advanced software.

### 2.7. TA Cloning Validation

The PCR products of 148 bp and 432 bp were purified and used for plasmid construction. TA cloning and Sanger sequencing were conducted by Invitrogen (Shanghai, China).

## 3. Results

### 3.1. The Principle of This Method for Haplotyping

**Figure 1:**
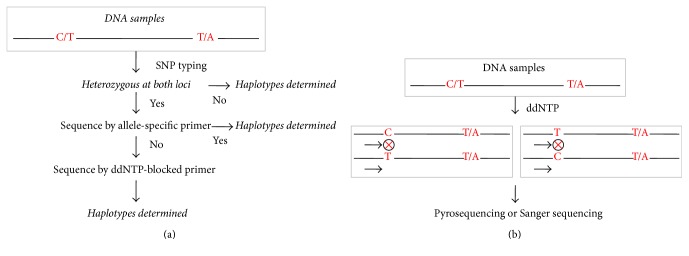
(a) Haplotype analysis flow diagram of PCR products sequenced by selective restriction of primer extension. First, single-stranded PCR products including two SNP loci were genotyped by pyrosequencing, and samples that were heterozygous at both loci were selected for further haplotype analysis. Allele-specific primers were then applied to haplotype by Sanger sequencing. If the samples were able to be sequenced with allele-specific primers, their haplotype could be determined. If not, they were further analyzed. Third, for the samples that could not be haplotyped with an allele-specific primer, specific ddNTP was added so as to selectively block sequencing primers during sequencing assays. (b) Principle of restricting the designated primer extension with ddNTP-blocked primers. A primer was first hybridized to a base at the front of the first heterozygous locus in the PCR products. When specific ddNTP was added, some of the primers were blocked during primer extension while others could not be blocked. The unblocked primers continued to extend. Finally, haplotypes were determined by pyrosequencing or Sanger sequencing.

**Figure 2:**
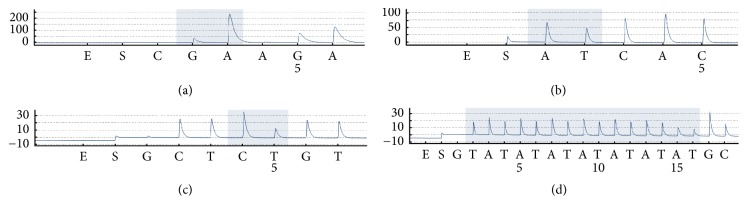
SNP genotyping results of PCR products. E means enzymes and S means substrates. The *y*-axis height of each peak (light signal) was proportional to the number of nucleotides incorporated and analyzed using the AQ mode of the PyroMark Q24 Advanced software. The 148-bp PCR products included two adjacent SNP sites (a) K1637K and (b) S1647T. The 432-bp PCR products included another two adjacent SNP sites (c)* UGT1A1∗*6 and (d)* UGT1A1∗*28.

Here, we have proposed a strategy for haplotype analysis of PCR products (Figure 1(a)). The procedures are as follows. First, single-stranded PCR products including two SNP loci were genotyped by pyrosequencing and Figure 2 shows their SNP genotypes when both loci were heterozygous with a content close to 50%. Second, samples that were heterozygous at both loci were selected for further haplotype analysis. Allele-specific primers were applied to haplotype by Sanger sequencing. If the samples were able to be sequenced with allele-specific primers, their haplotype could be determined. Third, for the samples that could not be haplotyped with the allele-specific primer, specific ddNTP was added so as to selectively block sequencing primers during sequencing assays. If the added ddNTP was complementary with DNA templates, the sequencing primers were blocked. If not, the unblocked primers would continue to extend, thus making haplotypes be determined by pyrosequencing or Sanger sequencing. A detailed haplotype analysis with ddNTP-blocked primers was shown in Figure 1(b). A primer was first hybridized to a base at the front of the first heterozygous locus in the PCR products. When specific ddNTP was added, some of the primers were blocked during primer extension while others could not be blocked. The unblocked primers continued to extend. Finally, haplotypes were determined by pyrosequencing or Sanger sequencing.

### 3.2. Haplotype Accuracy and Applicability of PCR Products Sequenced by Allele-Specific Primers

**Figure 3:**
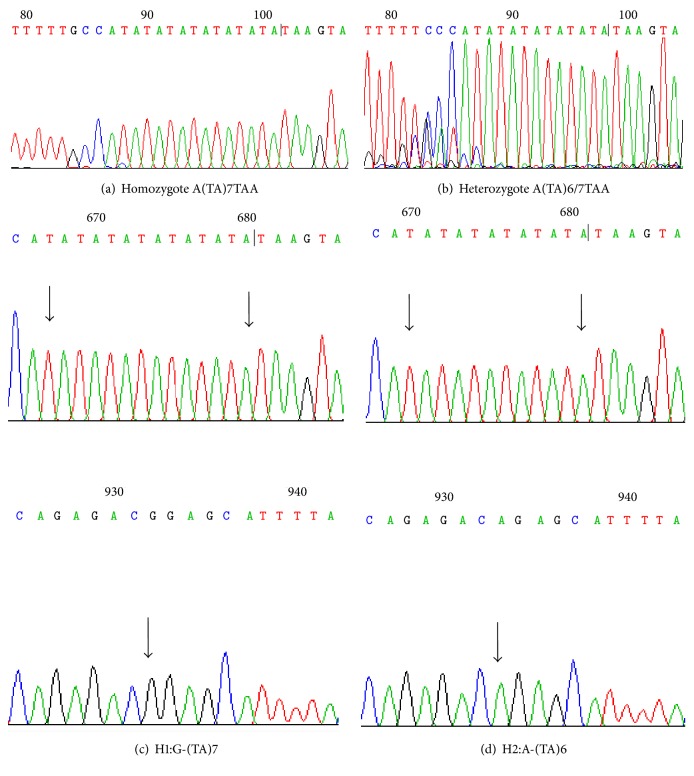
Sanger sequencing results (reverse complement) of PCR products including* UGT1A1∗*28 A(TA)6/7TAA and* UGT1A1∗*6 G>A. (a) Sequencing by the primer SP6. (b) Sequencing by the primer SP7. (c) and (d) were two haplotypes obtained from TA cloning and Sanger sequencing. The arrows indicated the loci tested.

For haplotype samples with adjacent loci that were both heterozygous, we first considered the AS-PCR method, which is a well known method for haplotyping [12, 13]. Selective amplification was achieved by designing a primer that matched/mismatched one of the alleles at the 3′*-end* of the primer. Therefore, based on the same principle, we first tried the method using allele-specific primer extension for haplotype analysis. SP6 and SP7 were allele-specific primers of the* UGT1A1∗*6 used to perform Sanger sequencing. The primer SP6 effectively limited the extension of the 3′*-end* mismatched C–A and obtained good typing results: Guanosine linked with A(TA)7TAA. For diploid biological samples, we could infer that the other haplotype was A–A (TA) 6TAA. TA cloning and Sanger sequencing showed that 14 constructed plasmids were H1 [G-(TA)7] and 16 plasmids were H2 [A-(TA)6] (Figure 3). The results indicated that the method was valid for PCR products containing two heterozygous SNPs (*UGT1A1∗*6 and* UGT1A1∗*28).

AS-PCR was one of the earliest methods used for haplotype analysis. However, it was labor-intensive and had a high cost when handling large populations. In our approach, SNP typing was applied and then the haplotype analysis was performed on two heterozygous loci with the following benefits. First, the haplotype of most samples could be determined by using only genotyping, greatly reducing the cost of analysis; second, using PCR products directly for further analysis could avoid not only the PCR primer design constraints but also error or failure caused by the AS-PCR.

**Figure 4:**
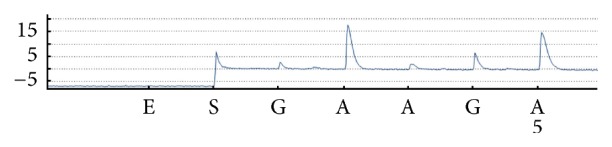
Pyrosequencing results of PCR products including K1637K A>G. Sequencing was conducted using the primer SP3 and template T1.

However, as reported in the literature, the biggest limitation of AS-PCR was that allele-specific primers can often be nonspecifically extended by most DNA polymerases, even if there was a mismatch with the template at its 3′-end, which resulted in false-positive results [14, 15]. Similarly, allele-specific primer extension sequencing may also have limitations. When using the primer SP7 for Sanger sequencing, the result was A(TA)7TAA/A(TA)6TAA heterozygous, which, according to this deduction, contained at least three haplotypes. This was contrary to common sense and indicated that the allele-specific primer SP7 sequencing result was not correct. SP7 was nonspecifically extended after the 3′-end T–G mismatch and produced double peaks. The same problem also existed in pyrosequencing. SP3 was an allele-specific primer of template T1 used to perform pyrosequencing. Nonspecific extension peaks could be observed for an error rate of over 8% compared with the normal control (Figure 4). Thermodynamic stability studies showed that mismatch types like “g:g,” “t:g,” “g:t,” “a:g,” and “g:a” were more stable than “t:t,” “a:a,” “c:t,” “t:c,” “c:a,” “a:c,” and “c:c” [16, 17]. Furthermore, the allele-specific primer extension was affected by the type, location, and number of mismatches [14].

### 3.3. Haplotype Analysis of PCR Products Sequenced by ddNTP-Blocked Primers

#### 3.3.1. Blocking Feasibility with ddNTPs

The most intuitive way to solve the problem caused by the above allele-specific primer extension sequencing was to avoid nonspecific extensions. The blocking efficiency was compared with that reported for three kinds of blocked primers: -Pi-, -NH_2_-, and ddNTP-modified primers in PCR amplifications mediated by Taq DNA polymerase and high-fidelity DNA polymerase [18]. Here, we used ddNTPs to block designated sequencing primers. The ddNTP-blocked primers cannot be extended by DNA polymerase because of the lack of free 3′-OH.

**Figure 5:**
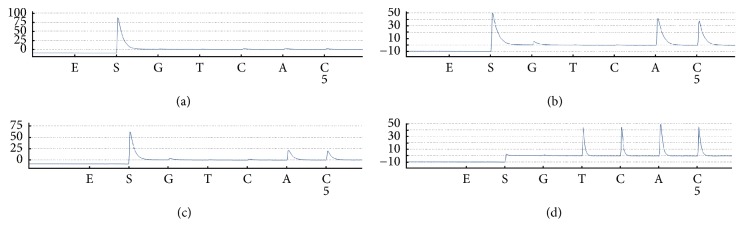
Pyrosequencing results of the primers were selectively blocked with ddTTP in exo^−^ Klenow polymerase. (a) Sequencing by the primer SP1 and synthesized template T1. (b) Sequencing by the primer SP1 and synthesized template T2. (c) Sequencing by the primer SP1 and an equal mixture of templates T1 and T2. (d) Sequencing by the primer SP1 and synthesized template T1 without adding ddTTP as a nonblocking control.

The extension of the ddNTP-blocked primers that needed to be inhibited effectively irrespective of which type of template was used. Therefore, the blocking efficiency of ddNTPs was the key factor affecting the specificity and accuracy of haplotype analysis. We performed pyrosequencing with a ddTTP-blocked hybridization mixture to verify the feasibility of this method. Synthesized T1 and T2 related to S1647T were applied as templates to hybridize with SP1. The results of raw data profiles showed that the extension of completely matched T1 could be well inhibited with ddTTP, while T2 containing a T–T mismatch extended normally (Figure 5). The feasibility experiment was carried out and the high substrate peak was caused by the undegraded pyrophosphate, indicating that the ddNTP synthesis reaction occurred. The substrate peak could be eliminated by adding suitable pyrophosphatase.

#### 3.3.2. Optimization of the Blocking Experimental Conditions

After verifying that ddNTP could restrict primer synthesis, 2 pmol and 4 pmol of primer SP1 were hybridized with 0.5, 1, 1.5, and 2 pmol template T1, respectively. The peak heights of all were about 40 and, therefore, were basically not different. Hence, more than 2 pmol of primers could be guaranteed to combine with a sufficient amount of template to produce a peak height suitable for observation. A sufficient amount of ddNTP was required to ensure that the incorporation reactions were complete. To determine the amount of ddNTP required, ddTTP at 1, 2, 4, 8, and 10 times the amount of the template were added for comparative experiments. The results indicated that when the amount of ddTTP was more than eight times the amount of the template, complete blocking was ensured in 40 minutes using exo^−^ Klenow.

The nucleotide incorporation reaction efficiency also depended on the DNA polymerase. Two different DNA polymerases were tested in this experiment: exo^−^ Klenow and Sequenase version 2.0 T7 DNA polymerase. Both DNA polymerases were exonuclease-free to prevent the removal of the ddNTP. However, exo^−^ Klenow incorporated ddNTP at a rate 1000 times slower than that for dNTP. Conversely, the ddNTP incorporation rate by T7 DNA polymerase was only slightly lower than that for dNTP [19]. After a comparative experiment, to achieve complete restriction, exo^−^ Klenow took 40 min, while T7 DNA polymerase only took 10 min.

According to the optimized results, we finally selected 4 pmol of the sequencing primer, 500 pmol of ddNTP, and 3.25 units Sequenase version 2.0 T7 DNA polymerase with the addition of pyrophosphatase for further study.

#### 3.3.3. Haplotype Accuracy of PCR Products Sequenced by ddNTP-Blocked Primers

**Figure 6:**
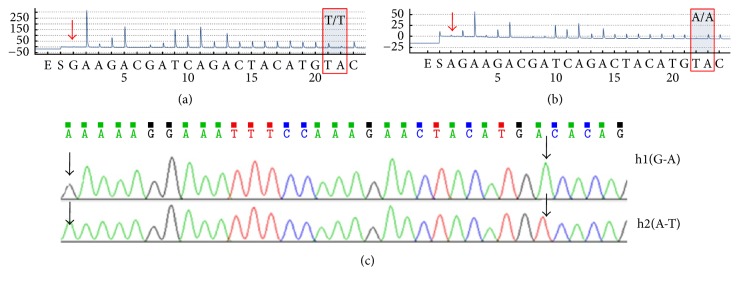
Pyrosequencing and Sanger sequencing results of PCR products including K1637K A>G and S1647T T>A. The red arrows indicated a ddNTP-blocked site. The black arrows represented SNP loci. The PCR products sequenced by the primer SP2 with the blocking of (a) ddGTP and (b) ddATP, respectively. (c) Two haplotypes obtained from TA cloning and Sanger sequencing.

K1637K and S1647T are located 27 bp apart: A/G AAAAG GAAAT TTCCA AAGAA CTACA TG A/T. Considering that the sequencing site K1637K is in the poly A position and that pyrosequencing has difficulty in reading the sequence in homopolymeric regions of the same base in DNA samples [20, 21], we optimized the dispensing order of dNTPs to ensure typing accuracy and sensitivity. Because the previous base of typing locus A/T was G, the GA bases after blocking locus A/G were added repeatedly to eliminate the effect of poly A in order to ensure that the typing result was correct. The programs of Figures 6(a) and 6(b) revealed that in the haplotypes of the selected PCR products, Adenine was linked with Thymine, and Guanine was linked with Adenine. Results of TA cloning (Figure 6(c)) indicated that two haplotypes were included in 30 constructed plasmids; they were h1 (G-A) and h2 (A-T), and the ratio was 13 : 17, respectively. The conclusion of the two typing methods was consistent.

**Figure 7:**
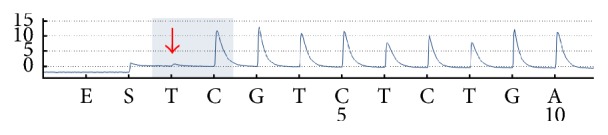
Pyrosequencing results of PCR products including* UGT1A1∗*6 G>A. Sequencing was conducted with the primer SP5 and ddTTP. The red arrow indicated the ddTTP-blocked site.

As seen from Figure 7, for the other two sites* UGT1A1∗*6 and* UGT1A1∗*28, that were 268 bp apart, we did not have Sanger sequencing equipment in the laboratory, while pyrosequencing was only suitable for analysis of short segment sequences; therefore, only the blocking feasibility by ddNTP of the relevant sites was verified by pyrosequencing.

The above experiments revealed that the haplotype analysis of PCR products was achieved by adding the blocking step, and the problem was solved when haplotype could not be analyzed by allele-specific primers.

## 4. Discussion

Haplotype-based association studies have been proposed as a powerful approach to disease gene mapping, based on linkage disequilibrium (LD) between causal mutations and the ancestral haplotypes by which they arose. For example, a number of mutations in the regulatory and coding regions of the* UGT1A1 *gene have been detected to confirm the diagnosis of Gilbert's syndrome [11, 22–24]. Haplotyping is usually conducted by indirect computational or direct experimental analysis. While being fast and of low cost, indirect methods are more susceptible to genotyping errors and missing data can have a significant effect on haplotype assignments. Computer simulation studies have identified that incorrect haplotype inferences typically occur for over 5% of the cases in a population of unrelated individuals [25]. The direct methods can give higher accuracy rate than indirect methods for each individual in a population, but they are slower, more labor-intensive, and costlier than the indirect methods.

Here, we have proposed a novel strategy for haplotype analysis from PCR products. The experimental results indicated that the haplotypes of PCR products,* UGT1A1∗*6 and* UGT1A1∗*28, were successfully analyzed by Sanger sequencing with the allele-specific primer extension. In addition, the haplotypes of PCR products, K1637K and S1647T, were used to verify pyrosequencing by restricting ddNTP-blocked primer synthesis. In this strategy, the PCR product was selected as the object of haplotype analysis, avoiding the design limitations of PCR primers. The haplotype of most samples can be determined with genotyping. Only two adjacent SNP loci that are both heterozygous need to be analyzed further. When a small number of samples need further analysis, subsequent PCR products of different samples at the same locus can be implemented. Allele-specific primers are attempted first. If it cannot be conducted, we choose ddNTPs to restrict primer extension to obtain the sequencing results.

This strategy has some advantages compared with existing experimental methods. For example, with AS-PCR performed directly using the genome as a template, each sample requires a separate two-step analysis, which increases the typing cost, and primer design may be limited. Furthermore, AS-PCR failure leads to erroneous results, and there are some analytic fragments that cannot be applied. Our previous method was the quantitative haplotyping of PCR products based on nonsynchronous pyrosequencing [9] and is more complex for the simple analysis of a sample from a single diploid organism. The coefficients for each haplotype need to be constructed, and they are only suitable for the analysis of short segment sequences. The sequencing process is troublesome, and the read length of the sequencing is still limited.

We originally wanted to apply selective ddNTP-blocking sequencing primers to Sanger sequencing for haplotyping of long segment PCR products. However, because of the limitation of pyrosequencing read length and lack of a Sanger sequencing instrument, haplotyping for a long PCR segment was not attempted by ddNTP-blocked primers, but it is theoretically feasible. Extension products of unblocked sequencing primers can be directly analyzed by Sanger sequencing to obtain the haplotype of the long PCR fragment. In principle, if there is 1 pmol of the PCR product, assuming that the sequence length is 1000 bp and the amount of ddNTP is approximately 1/1000 of dNTP, the number of first labeled fragments is: 10^−12 ^mol × 0.5 × 6 × 10^23^ mol^−1^ × 1/1000 = 3 × 10^8^ molecules, while the amount of the 1000th labeled fragments is 3 × 10^5^ molecules. Therefore, it is feasible to analyze the extension products of specific sequencing primers directly using a simple method by Sanger sequencing.

In conclusion, we have developed a novel sequencing strategy for molecular haplotyping of unrestricted conventional PCR products from natural samples. This method is simple, fast, and unaffected by the read length restriction of pyrosequencing. It may provide a precise and fast sample-to-answer system for haplotypic determination in clinical diagnosis.

## Figures and Tables

**Table 1:** Nine possible haplotypes at two loci in diploid organisms.

First locus	A/A	A/T	T/T
Haplotype			
Second locus			
G/G	A—G; A—G	A—G; T—G	T—G; T—G
G/C	A—G; A—C	A—G; T—C or A—C; T—G	T—G; T—C
C/C	A—C; A—C	A—C; T—C	T—C; T—C

**Table 2:** Synthesized sequences for verifying the feasibility of ddNTP blocking efficiency.

Name	Nucleotide sequence (5→3)
T1	Biotin-TAAAATACTGTG***A***CATGTAGTTCTTTGGAAATTTCCTTTT***T***TTTGAAAGAAATTTTTCCACATCTCTACGCGAAA
T2	Biotin-TAAAATACTGTG***T***CATGTAGTTCTTTGGAAATTTCCTTTT***C***TTTGAAAGAAATTTTTCCACATCTCTACGCGAAA
SP1	AAAT TTCCAAAGAA CTACATG

T1 and T2 represented synthesized haplotypes related to K1637K and S1647T. The italic segments were the polymorphic sites, and the underlined segments were the regions that hybridized with the sequencing primer SP1.

**Table 3:** Synthesized sequences used for genotyping and haplotyping.

Genotype	PCR primer sequences (5′→3′)	Typing primer sequences (5′→3′)	Typing primer applications
S1647T (T>A)	TF: TGACAGTGAAAGTGGAAGGTTG	SP1: AAATTTCCAAAGAACTACATG	Genotyping

K1637K (A>G)	TR: Biotin-CCTATTGGCAAAGCAATCTGGA	SP2: TGTGGAAAAATTTCTTTCAAA	Genotyping; haplotyping^*∗*^
		SP3: TGTGGAAAAATTTCTTTCAAAG	Haplotyping^*∗∗*^

*UGT1A1∗*28 [A(TA)6TAA>A(TA)7TAA]	GF: Biotin-CCCTGCTACCTTTGTGGACT	SP4: GGTTCGCCCTCTCCTACTTA	Genotyping

*UGT1A1∗*6 (G>A)	GR: CATTATGCCCGAGACTAACAAA	SP5: GTCTTCAAGGTGTAAAATGCTC	Genotyping; haplotyping^*∗*^
		SP6: GTCTTCAAGGTGTAAAATGCTCC	Haplotyping^*∗∗*^
		SP7: GTCTTCAAGGTGTAAAATGCTCT	Haplotyping^*∗∗*^

The SNP sites associated with typing primers were as follows. S1647T: SP1; K1637K: SP2 and SP3; *UGT1A1∗*28: SP4; *UGT1A1∗*6: SP5, SP6, and SP7. ^*∗*^Based on ddNTP blocking. ^*∗∗*^Based on allele-specific primer extension.
